# *FMR1* allelic complexity in premutation carriers provides no evidence for a correlation with age at amenorrhea

**DOI:** 10.1186/s12958-024-01227-5

**Published:** 2024-06-21

**Authors:** Bárbara Rodrigues, Vanessa Sousa, Carolyn M. Yrigollen, Flora Tassone, Olatz Villate, Emily G. Allen, Anne Glicksman, Nicole Tortora, Sarah L. Nolin, António J. A. Nogueira, Paula Jorge

**Affiliations:** 1 Molecular Genetics Laboratory, Laboratory Genetics Service, Genetics and Pathology Clinic, Unidade Local de Saúde de Santo António (ULSSA), Porto, Portugal; 2 UMIB - Unit for Multidisciplinary Research in Biomedicine, ICBAS – School of Medicine and Biomedical Sciences, UPorto – University of Porto, Porto, Portugal; 3 ITR – Laboratory for Integrative and Translational Research in Population Health, Porto, Portugal; 4 Present Address: Cytogenetics Laboratory, Department of Microscopy, ICBAS – School of Medicine and Biomedical Sciences, UPorto – University of Porto, Porto, Portugal; 5 Raymond G. Perelman Center for Cellular and Molecular Therapeutics, Children’s Hospital of Philadelphia Research Institute, Philadelphia, PA USA; 6 Department of Biochemistry and Molecular Medicine, School of Medicine, University of California, Davis, Stockton Blvd, USA; 7 MIND Institute, School of Medicine, University of California, Davis, CA USA; 8 Pediatric Oncology Group, Biocruces Bizkaia Health Research Institute, Barakaldo, Biscay, Basque Country, Spain; 9 Department of Human Genetics, Emory University School of Medicine, Atlanta, GA USA; 10 New York State Institute for Basic Research in Developmental Disabilities, New York, NY USA; 11 CESAM – Center for Environmental and Marine Studies, Department of Biology, University of Aveiro, Aveiro, Portugal

**Keywords:** *FMR1* gene premutation, Age at amenorrhea, *FMR1* allelic complexity, Fragile X-associated primary ovarian insufficiency, CGG repeats, AGG interspersion pattern

## Abstract

**Background:**

Premutations in the Fragile X Messenger Ribonucleoprotein 1 (*FMR1*) gene, defined as between 55 and 200 CGGs, have been implicated in fragile X-associated primary ovarian insufficiency (FXPOI). Only 20% of female premutation carriers develop early ovulatory dysfunction, the reason for this incomplete penetrance is unknown. This study validated the mathematical model in premutation alleles, after assigning each allele a score representing allelic complexity. Subsequently, *allelic scores* were used to investigate the impact of allele complexity on age at amenorrhea for 58 premutation cases (116 alleles) previously published.

**Methods:**

The *allelic score* was determined using a formula previously described by our group. The impact of each *allelic score* on age at amenorrhea was analyzed using Pearson’s test and a contour plot generated to visualize the effect.

**Results:**

Correlation of *allelic score* revealed two distinct complexity behaviors in premutation alleles. No significant correlation was observed between the *allelic score* of premutation alleles and age at amenorrhea. The same lack of significant correlation was observed regarding normal-sized alleles, despite a nearly significant trend.

**Conclusions:**

Our results suggest that the use of *allelic scores* combination have the potential to explain female infertility, namely the development of FXPOI, or ovarian dysfunction, despite the lack of correlation with age at amenorrhea. Such a finding is of great clinical significance for early identification of females at risk of ovulatory dysfunction, enhancement of fertility preservation techniques, and increasing the probability for a successful pregnancy in females with premutations. Additional investigation is necessary to validate this hypothesis.

**Supplementary Information:**

The online version contains supplementary material available at 10.1186/s12958-024-01227-5.

## Background

The *Fragile X Messenger Ribonucleoprotein 1* (*FMR1*) gene, located on the X chromosome (Xq27.3), contains a polymorphic CGG repeat on its 5’ untranslated region (UTR) implicated in three disorders depending on the repeat number: Fragile X Syndrome (FXS; OMIM #300624) when CGGs > 200, and Fragile X-associated Tremor/Ataxia Syndrome (FXTAS; OMIM #300623) and Fragile X-associated Primary Ovarian Insufficiency (FXPOI; OMIM #311360) [1–3] in the premutation range 55 < CGG < 200. The mechanism of FXPOI development is not fully understood but it is believed to be due to the toxic effect of elevated *FMR1* mRNA levels [2, 4]. Premutation carriers with FXPOI show hypergonadotropic hypogonadism and absent or irregular menstrual cycles before 40 years of age [5]. The CGG repeat length correlates unevenly with FXPOI, as females carrying 70 to 100 CGGs have an increased risk of FXPOI when compared with those with more than 100 repeats [6, 7]. Furthermore, FXPOI development is not fully penetrant. In *FMR1* normal-sized alleles (5 to 44 CGGs), the repetitive region is usually interrupted by one or more AGGs, typically occurring at every 9^th^ or 10^th^ CGG [8]. Premutation alleles are predominantly composed of pure CGGs; loss of AGG interruption(s) has been linked to the instability of the repetitive region and the increased risk of expansion [3, 9–13]. A formula integrating the total repeat length, and the number and pattern of the AGGs was developed to calculate *FMR1 allelic score* [14]. *Allelic score* is a metric that reflects the complexity of the *FMR1* gene CGG repetitive region. Herein, we evaluate the association between the combination of normal and premutation *allelic scores* and ovulatory dysfunction underlying FXPOI. Our formula was applied to calculate the *allelic scores* in *FMR1* premutation carriers, validating its use in samples with distinct genotypic characteristics. It was hypothesized that the combination of the AGG number and pattern from both normal and premutation alleles would associate indirectly with age at amenorrhea and hormone levels with a potential impact on FXPOI development.

## Methods

### *FMR1 allelic scores* determination

Molecular data of both alleles regarding samples from premutation carriers, previously published, were requested to the respective authors: Villate et al. [15] (Spain), Allen et al. [16] (United States of America) and Yrigollen et al. [17] (United States of America). Of all data provided by the authors, 577 results were retrieved: Villate et al. [15] (*n* = 20, designated by set 1), Allen et al. [16] (*n* = 59, designated by set 2) and Yrigollen et al. [17] (*n* = 498, designated by set 3). The *allelic score*, which reflects the *FMR1* CGG/AGG substructure, was calculated separately for each allele (normal and premutation), using the formula described in Rodrigues et al. [14]. The age at amenorrhea - defined by at least 4 months of secondary amenorrhea and menopausal levels of follicle-stimulating hormone (FSH) [16] - was reported in 58 observations from set 2 (mean age 38.7 ± 8.5 years, range 18–56) thus resulting in a slightly smaller dataset (58 observations instead of 59).

### Reference set

The reference set, composed of one hundred and thirty-one female samples with normal (*n* = 127) and intermediate genotypes (*n* = 4), was previously described and characterized in Rodrigues et al. [14] (the summary of the results can be found in Supplementary Table 1).

### Statistical analysis

A linearized form of a logarithmic model [i.e., regression of ln(score 1) against score 2] was used to obtain a functional model to relate the complexity of both alleles in each set. Covariance analysis (ANCOVA) compared the reference set with premutation sample set regression models, following the methodology outlined by Zar [18]. SigmaPlot version 14.0 (Systat Software®Inc., Chicago, IL, USA) was used for One-Way ANOVA on ranks (Kruskal-Wallis test) to compare separately *allelic score* and the size of each allele (normal and premutation). Dunn’s method was used for multiple comparisons after conducting a Kruskal-Wallis test, comparing sets based on median allele size and *allelic score*. The relationship between the age at amenorrhea and *allelic score* was assessed by Pearson correlation coefficient. R sofware version 4.3.0 by R Core Team [19] with the ggplot2 package [20] was used to generate contour plots to display the relationship between independent variables normal and premutation *allelic scores*, and the dependent variable, age at amenorrhea. All statistical tests were carried out for a significance level of 0.05.

## Results

### *FMR1* CGG repeat characterization

*FMR1* molecular data of 1154 alleles are summarized in Supplementary Table 2. In a set 3 sample both alleles are in the premutation (PM) range, a rare event previously reported in seven cases [21]. The most frequent repeat length among normal-sized alleles is 30 CGGs, despite the significant differences among allele sizes (Kruskal-Wallis test: H = 12.3; df = 2; *p* = 0.002) (Supplementary Fig. 1a). The majority of normal-sized alleles contained one or two AGG interruptions (93.8%, *n* = 540) while pure alleles occurred in 4.7% of samples (*n* = 4, set 2, *n* = 23, set 3), and the remaining 1.5% of samples showed three AGGs (*n* = 9, set 3). In total, ninety-seven different AGG patterns (*n* = 8/ 20, set 1, *n* = 23/ 59, set 2 and *n* = 75/ 498, set 3) were identified. The most common AGG interspersion pattern in sets 1 and 3 is (CGG)_10_AGG(CGG)_9_AGG(CGG)_9_. On the contrary, a very rare pattern was identified as commonest among set 2 samples (CGG)_11_AGG(CGG)_10_AGG(CGG)_7_. Around half of the PM alleles had no AGGs (50.3%, *n* = 8, set 1, *n* = 26, set 2, *n* = 257, set 3), and approximately 49.7% showed one or two AGG interruptions (*n* = 287). Two hundred and eleven different patterns were identified (*n* = 13/ 20, set 1, *n* = 45/ 59, set 2 and *n* = 177/ 498, set 3) in PM alleles revealing very exclusive CGG/AGG structures.

### Mathematical model validation

**Fig. 1:**
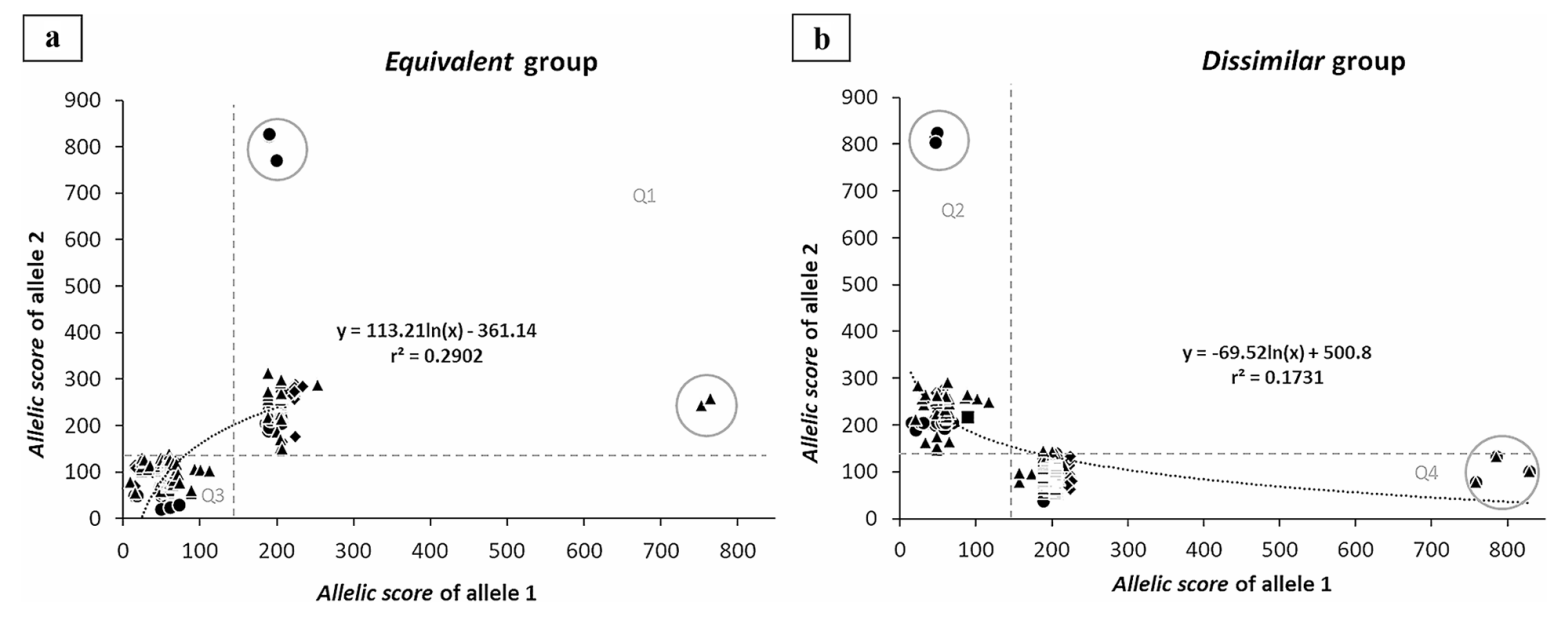
Correlation between the *FMR1* allelic complexity (*allelic score*) of each allele in all sets, according to groups: *equivalent* (**a**) and *dissimilar* (**b**). The graph was partitioned into four quadrants based on the dispersion of the data: Q1 (quadrant 1), Q2 (quadrant 2), Q3 (quadrant 3), and Q4 (quadrant 4). The reference set is denoted by circle symbols, while set 1, set 2, and set 3 are represented by squares, lozenges, and triangles, respectively. Samples marked with a grey circle represent alleles with an *allelic score* above 700

Descriptive statistics and frequency analyses of premutation *allelic scores* are shown in Table 1. The median PM *allelic scores* did not show statistically significant differences between sets (Kruskal - Wallis test: H = 1.45; df = 2; *p* = 0.484) (Supplementary Fig. 1d); despite the sets having significantly different normal median *allelic scores* (Kruskal-Wallis test: H = 33.20; df = 2; *p* < 0.001) (Supplementary Fig. 1b). To compare these PM samples with previously published data using the same mathematical model, a reference set was built from that publication [14]. All sets distributed *allelic scores* into four quadrants, separated by a value of 150 as previously observed in the reference set, revealing similar compositions (Fig. 1): samples with alleles showing a similar complexity (*equivalent* group, quadrants 1 and 3) (Fig. 1a) and samples where alleles have a different complexity (*dissimilar* group, quadrants 2 and 4) (Fig. 1b). Thus, the correlation between the *allelic score* of each allele (Fig. 1a and b) was described following a logarithmic model. Significant correlations were found in both groups from all sets: reference set – *equivalen*t group: *r* = 0.551; df = 71; *p* < 0.0001 and *dissimilar* group: *r* = 0.466; df = 54; *p* < 0.0001 (Fig. 1a and b, represented by circles); set 1 – *equivalen*t group: *r* = 0.994; df = 8; *p* < 0.0001 and *dissimilar* group: *r* = 0.991; df = 6; *p* < 0.0001 (Fig. 1a and b, represented by squares); set 2 – *equivalent* group *r* = 0.933; df = 26; *p* < 0.0001 and *dissimilar* group: *r* = 0.938; df = 27; *p* < 0.0001 (Fig. 1a and b, represented by lozenges), and set 3 – *equivalent* group: *r* = 0.912; df = 187; *p* < 0.0001 and *dissimilar* group: *r* = 0.882; df = 297; *p* < 0.0001 (Fig. 1a and b, represented by triangles). An exponential growth of the *allelic score* was observed, particularly in alleles having more than two AGGs (Supplementary Table 3); due to the relevance attributed to the AGG number by the formula. For instance, samples with three AGGs show scores above 700 (*n* = 6, reference set, and *n* = 8, set 3; represented by a grey circle in Fig. 1a and b). To validate the mathematical model in expanded alleles a covariance analysis between the reference and PM sample sets logarithmic models was performed separately for each group (Supplementary Fig. 2). Supplementary Table 4 shows the individual models resulting from each set. Coincident regression lines demonstrate the absence of statistically significant differences in each *equivalent* and *dissimilar* groups from PM samples sets when compared with those of the reference set. This result supports a more robust model including observations from the four sets: *equivalent* group – F _(6, 300)_ = 1.8278; *p* = 0.0934: Score 2 = -238.3 + 87.4 × ln(score 1) and *dissimilar* group – F _(6, 392)_ = 1.0679; *p* = 0.3812: Score 2 = 573.9–88.4 × ln(score 1).

**Table 1:** Summary of the *FMR1* allelic complexity (*allelic score*) results

		A1 - Shorter CGG repeat length allele			A2 - Longer CGG repeat length allele		
		Set 1	Set 2	Set 3	Set 1	Set 2	Set 3
Number of alleles		40	118	996	40	118	996
*Allelic score*	Mean (± S.D.)	159.0 ± 66.5	165.8 ± 79.8	159.7 ± 105.5	152.7 ± 81.8	150.2 ± 81.7	131.0 ± 61.6
	Median	205	207	193	217	214	109
	Range	49–206	16–234	9–829	56–242	63–288	55–313
	Most frequent (%, n)	205 (30%, *n* = 6)	223 (27.1%, *n* = 16)	205 (34.9%, *n* = 174)	231 (15%, *n* = 3)	133 (5.1%, *n* = 3)	103 (2.4%, *n* = 12)
		206 (25%, *n* = 5)	207 (18.6%, *n* = 11)	189 (12.7%, *n* = 63)	217 (15%, *n* = 3)	83 (5.1%, *n* = 3)	100 (2.4%, *n* = 12)
*Allelic scores* Kruskal-Wallis Test		H = 33.20; df = 2; *p* < 0.001			H = 1.45; df = 2; *p* = 0.484		

S.D. = Standard deviation; % = Frequency; n = Number of alleles

*p* values represent significant levels between sets 1, 2 and 3 *allelic scores*; Multiple Comparison (Dunn’s Method) results in Supplementary Fig. 1b and d

Data published in Villate et al. [15] (set 1), Allen et al. [16] (set 2) and Yrigollen et al. [17] (set 3)

### *FMR1 allelic scores* and age at amenorrhea association

To understand the impact of *FMR1 allelic score* on the age at amenorrhea, normal-sized (allele 1) and PM (allele 2)alleles from set 2 samples were analyzed separately (*n* = 58). No significant correlation was observed between A1 *allelic score* and age at amenorrhea (*p* > 0.05) (Supplementary Fig. 3a, c, e and g). The same was true when PM *allelic score* was used (*p* > 0.05) (Supplementary Fig. 3b, d, f, and h). A nearly significant trend (*p* = 0.058) is apparent between the A1 *allelic score* and age at amenorrhea in samples showing an *allelic score* between 206 and 234 (Supplementary Fig. 3a) and 16–68 (Supplementary Fig. 3e) (quadrants 1 and 3, respectively). Two distinct behaviors were observed: age at amenorrhea rise with increasing *allelic score* (above 200, quadrant 1, mean age at amenorrhea 40 ± 8.5 years, Supplementary Fig. 3a), and age at amenorrhea decrease with increasing *allelic score* (below 70, quadrant 3, mean age at amenorrhea 38 ± 8 years, Supplementary Fig. 3e). The majority of these samples have alleles with less than 26 CGGs (78.6%, *n* = 11), with one or no AGG interspersions (71.4%, *n* = 10, 28.6%, *n* = 4, respectively), whereas those with higher *allelic scores* have alleles ranging from 29 to 32 CGGs, with two AGG interspersions.

### Age of amenorrhea assessment by *allelic scores combination*

**Fig. 2:**
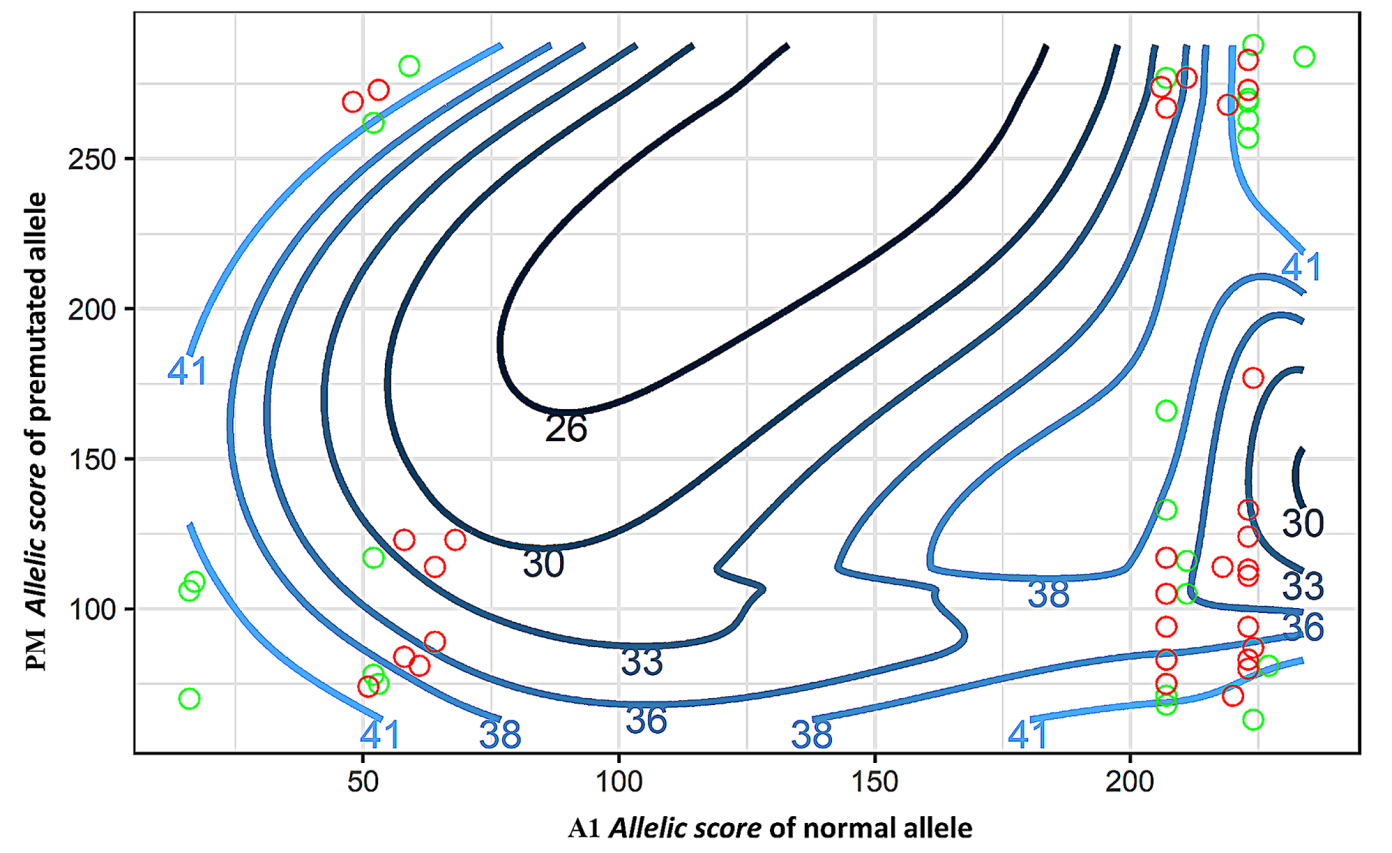
Contour plot evaluating the interaction between three variables: *allelic score* of both alleles (independent variables – x, y) and age of amenorrhea (dependent variable – z). Samples in witch combination of allelic complexity is associated with amenorrhea before age 40 years and after age 40 years are represented by red and green circles, respectively. The different colors of the contour lines indicate the ages at amenorrhea as a function of the *allelic score* of both alleles

PM alleles within the range 70–100 CGGs are known to have increased risk of developing FXPOI [6, 7, 22], however not all carriers develop disease and there is lack of knowledge on the underlying mechanisms. This led us to speculate if FXPOI development could be associated with a combined effect of *FMR1* allelic complexity. To analyze the joint effect of A1 and PM *allelic scores* in the age at amenorrhea, a contour plot was generated. Overall, different trends were observed: menopause age approaches normal (mean 51 years, range 40 to 60 years) when the *allelic score* of both alleles increases or decreases, showing that balanced *allelic scores* have minimal impact on early amenorrhea. Deeper analysis of samples with mean age at amenorrhea below 40 years and PM *allelic score* between 70 and 123 show that age decreases with increasing A1 *allelic score* (Fig. 2, A1 *allelic score* between 50 and 55).

## Discussion

In our study, aiming to validate the previously published mathematical model ascertained in normal and intermediate alleles, we compared three distinct datasets with premutation carriers and subsequently explored the relationship between allelic complexity of the *FMR1* gene and age at amenorrhea – a clinical manifestation associated with development of FXPOI.

A comparative analysis of the CGG/AGG substructure across the sets revealed that the normal-sized allele with 30 repeats was the most frequent with the more prevalent AGG interspersion pattern being (CGG)_10_AGG(CGG)_9_AGG(CGG)_9_ in sets 1 and 3, consistent with findings in other populations [14, 23, 24]. A notably rare pattern, (CGG)_11_AGG(CGG)_10_AGG(CGG)_7_, emerged as the most prevalent in set 2, which can be attributed to intrinsic characteristics of this subpopulation such as complexity and heterogeneity of genetic traits. Expectedly, approximately half of the PM alleles showed one or two AGG interruptions with an overall average repeat length of 90.3 (S.D. = 20.8), since PM alleles with longer CGG lengths tend to demonstrate a lower incidence of AGG interruptions [11]. Notably, many different AGG interspersion patterns were found in PM alleles, most probably due to the inherent instability of these alleles [8].

No statistically significant difference was observed among the *allelic score* of PM alleles, but was found for the *allelic score* of normal-sized alleles. A similar result was observed when comparing the total CGG repeat length of normal alleles, revealing great variability in the complexity of the CGG repetitive region of normal-sized alleles. The combination of *allelic scores* revealed the emergence of two groups with distinct characteristics: *equivalent* and *dissimilar*, both exhibiting significant correlations. Similar outcomes had been reported by Rodrigues et al. [14]. The validation of our mathematical model in females with *FMR1* expansions showed that this model can be applied in populations that exhibit varied genotypic characteristics, namely expanded alleles such as premutations.

Several studies have sought to comprehend the impact of the CGG repetitive region on the development of FXPOI. However, the majority focus on examining the influence of the CGGs and AGGs independently, as exemplified by Friedman-Gohas et al. study [25]. Here, we employed our formula, which integrate the total CGG repeat length, the number of AGGs, and the AGG interspersion pattern. No statistically significant correlations were found between A1 *allelic scores* and age at amenorrhea, nor PM *allelic scores* and age at amenorrhea. The lack of statistical significance might be due to the reduced number of observations in each grouping [26]. Nevertheless, a significant trend was observed with normal *allelic scores* between 206 and 234 and 16–68 and age at amenorrhea. The influence of *FMR1* gene alleles within normal size in ovarian reserve is controversial. Gleicher et al., demonstrated a negative effect in ovarian reserve of alleles with less than 26 CGGs, evidenced by low levels of anti-Müllerian hormone (AMH) [27–29]. Wang et al. demonstrated reduction in *FMR1* mRNA levels in granulosa cells from females carrying alleles with CGGs < 26 and simultaneously a misregulation steroidogenic enzymes and hormone receptors, leading to ovarian dysfunction and ultimately infertility [30]. Rechnitz et al. illustrated a poor response to ovarian stimulation and elevated expression of *FMR1* mRNA in granulosa cells when compared to samples with different *FMR1* gene sub-genotypes [31]. Interestingly, the majority of our samples with alleles < 26 CGGs show one or no AGGs, while alleles with a repeat size between 29 and 32 CGGs show two AGG interruptions. Lekovich et al. demonstrated that premutation with none or one AGG showed poorer ovarian reserve than those with two, suggesting AGG interspersions have a protective effect [32]. It is thus tempting to speculate that by a similar mechanism the absence of AGGs in normal alleles correlates with ovarian dysfunction.

A minimal effect of *FMR1* allelic complexity with age at amenorrhea is observed in balanced *allelic scores*. Moreover, it appears that the age of amenorrhea decreased with increasing A1 *allelic score* when the PM allele had a score between 70 and 123. Despite the absence of statistical significance, a trend towards a correlation with the *allelic score* of the A1 suggests the need for larger-scale investigations to assess the impact of the combined *allelic scores* on the age at amenorrhea. It is likely that the age at amenorrhea may not provide a comprehensive assessment of FXPOI development. Therefore, it is important to test other clinical parameters, such as AMH levels, to gain a deeper understanding of the impact of combining *allelic scores* on disease development.

## Conclusion

This is the first report investigating the combined effect of normal and premutation *allelic scores* on FXPOI development impacted by age at amenorrhea. In our analysis, the presence of the correlation trend indicates the need for further studies and additional samples to explore the complex relationship between *allelic score* combinations and the development of FXPOI. Skewed X chromosome inactivation and hormonal deregulation were not considered and might impact age at amenorrhea. Nevertheless, the use *allelic scores* combination may pave the way to the identification of an ovulatory dysfunction biomarker. This is of major clinical importance to improve fertility in premutation carriers, to make choices about preservation strategies such as oocyte cryopreservation, increasing chances of a successful pregnancy.

### Electronic supplementary material

Below is the link to the electronic supplementary material.

Supplementary Material 1

Supplementary Material 2

## Data Availability

Data is contained within the article or supplementary material.

## Abbreviations

AGG: Adenine-Guanine-Guanine
AMH: anti-Müllerian hormone
CGG: Cytosine-Guanine-Guanine
*FMR1*: *fragile x messenger ribonucleoprotein 1* gene
FSH: Follicle-stimulating hormone
FXPOI: Fragile X-associated Primary Ovarian Insufficiency
FXS: Fragile X Syndrome
FXTAS: Fragile X-associated Tremor/Ataxia Syndrome
PM: Premutation
