# Supplementary material for: FMR1 allelic complexity in premutation carriers provides no evidence for a correlation with age at amenorrhea
Source: Reprod Biol Endocrinol. 2024 Jun 21;22:71. doi: 10.1186/s12958-024-01227-5 (PMC11191145; doi:10.1186/s12958-024-01227-5)
Supplement: Supplementary file 1 — Supplementary Material 1 [file 12958_2024_1227_MOESM1_ESM.docx]

***FMR1* allelic complexity in premutation carriers provides no evidence for a correlation with age at amenorrhea**

**Bárbara Rodrigues**

Molecular Genetics Laboratory, Laboratory Genetics Service, Genetics and Pathology Clinic, Unidade Local de Saúde de Santo António (ULSSA), Porto, Portugal,

UMIB – Unit for Multidisciplinary Research in Biomedicine, ICBAS – School of Medicine and Biomedical Sciences, UPorto - University of Porto, Porto, Portugal

ITR – Laboratory for Integrative and Translational Research in Population Health, Porto, Portugal

ID: 0000-0003-4698-962X

**Vanessa Sousa**

Molecular Genetics Laboratory, Laboratory Genetics Service, Genetics and Pathology Clinic, Unidade Local de Saúde de Santo António (ULSSA), Porto, Portugal

Current address: Cytogenetics Laboratory, Department of Microscopy, ICBAS – School of Medicine and Biomedical Sciences, UPorto – University of Porto, Porto, Portugal

UMIB – Unit for Multidisciplinary Research in Biomedicine, ICBAS – School of Medicine and Biomedical Sciences, UPorto - University of Porto, Porto, Portugal

ITR – Laboratory for Integrative and Translational Research in Population Health, Porto, Portugal

ID: 0000-0002-5855-1863

**Carolyn M. Yrigollen**

Raymond G. Perelman Center for Cellular and Molecular Therapeutics, Children’s Hospital of Philadelphia Research Institute, Philadelphia, PA, USA

ID: 0000-0002-2399-6176

**Flora Tassone**

Department of Biochemistry and Molecular Medicine, University of California, Davis, School of Medicine, Stockton Blvd, USA

MIND Institute, University of California, Davis, School of Medicine, Davis, CA, USAID: 0000-0002-6388-9180

**Olatz Villate**

Pediatric Oncology Group, Biocruces Bizkaia Health Research Institute, Barakaldo, Biscay, Basque Country, Spain

ID: 0000-0002-5574-837X

**Emily G. Allen**

Department of Human Genetics, Emory University School of Medicine, Atlanta, GA, USA

ID: 0000-0003-0963-036X

**Anne Glicksman**

New York State Institute for Basic Research in Developmental Disabilities, New York, NY, USA

**Nicole Tortora**

New York State Institute for Basic Research in Developmental Disabilities, New York, NY, USA

**Sarah L. Nolin**

New York State Institute for Basic Research in Developmental Disabilities, New York, NY, USA

ID: 0000-0001-5209-289X

**António J. A. Nogueira**

CESAM – Center for Environmental and Marine Studies, Department of Biology, University of Aveiro, Aveiro, Portugal

ID: 0000-0001-7089-2508

**Paula Jorge**

Molecular Genetics Laboratory, Laboratory Genetics Service, Genetics and Pathology Clinic, Unidade Local de Saúde de Santo António (ULSSA), Porto, Portugal

Current address: Cytogenetics Laboratory, Department of Microscopy, ICBAS – School of Medicine and Biomedical Sciences, UPorto – University of Porto, Porto, Portugal

UMIB – Unit for Multidisciplinary Research in Biomedicine, ICBAS – School of Medicine and Biomedical Sciences, UPorto - University of Porto, Porto, Portugal

ITR – Laboratory for Integrative and Translational Research in Population Health, Porto, Portugal

ID: 0000-0002-6507-222X

**Corresponding author**

**Paula Maria Vieira Jorge**

E-mail: [paulajorge.cgm@chporto.min-saude.pt](mailto:paulajorge.cgm@chporto.min-saude.pt)

Contact: 22 607 0330

**Additional file 1.** Supplementary figures.


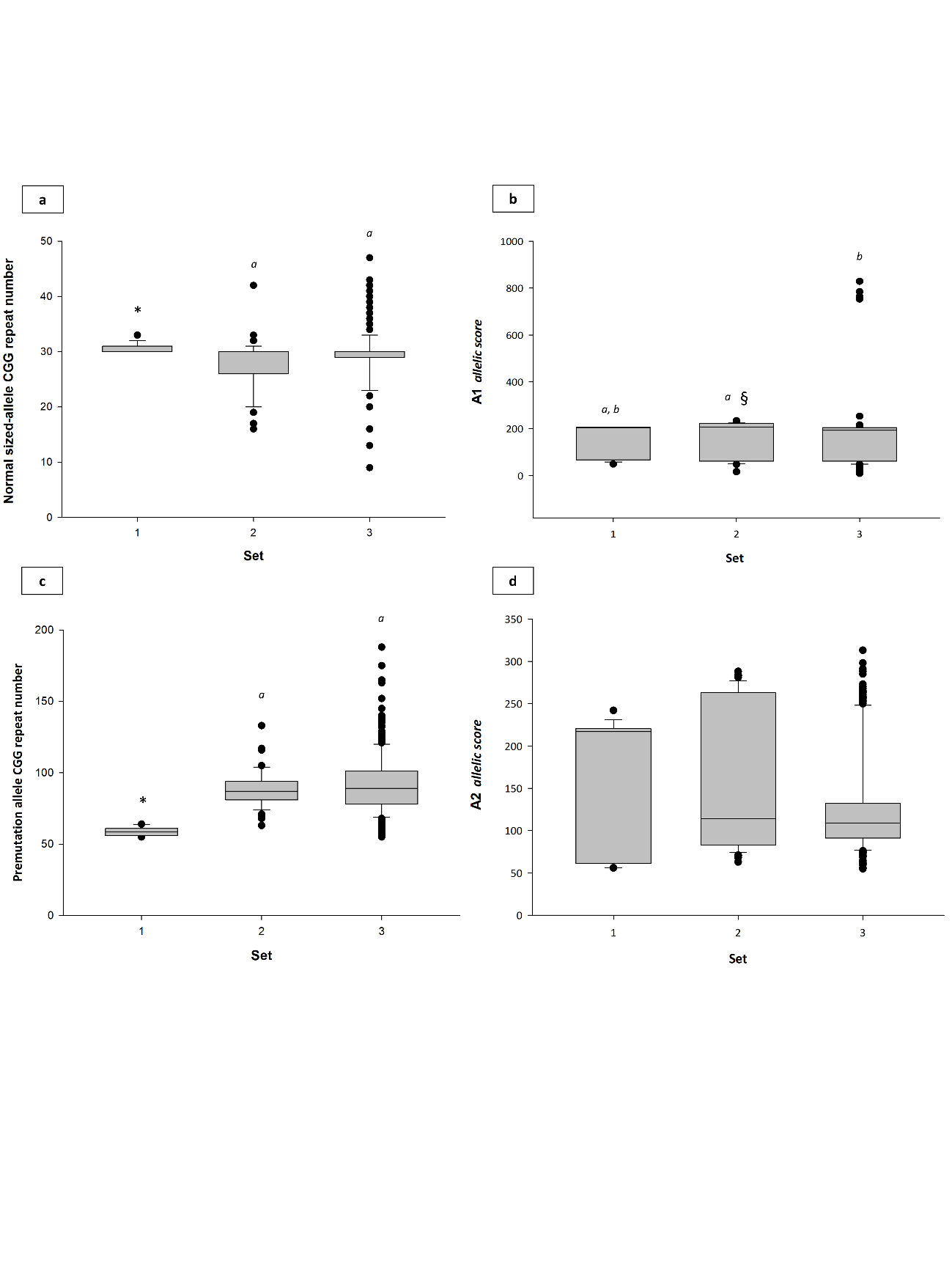


**Supplementary Fig. 1** CGG repeat number (a), (c) and *allelic scores* (b), (d) distribution. A1 - Shorter CGG repeat length allele; A2 - Longer CGG repeat length allele; ^*^Statistically significant differences were found in both alleles from set 1 when compared with sets 2 and 3 (Dunn's Method, normal-sized alleles: *p* = 0.02 and *p* = 0.016 respectively; premutation alleles: *p* < 0.001); ^§^Set 2 *allelic score* is statistically different from set 3 (Dunn's Method: *p* < 0.001); *^a, b^*No statistically significant differences (Dunn's Method: *p* > 0.05). See Supplementary Table 2


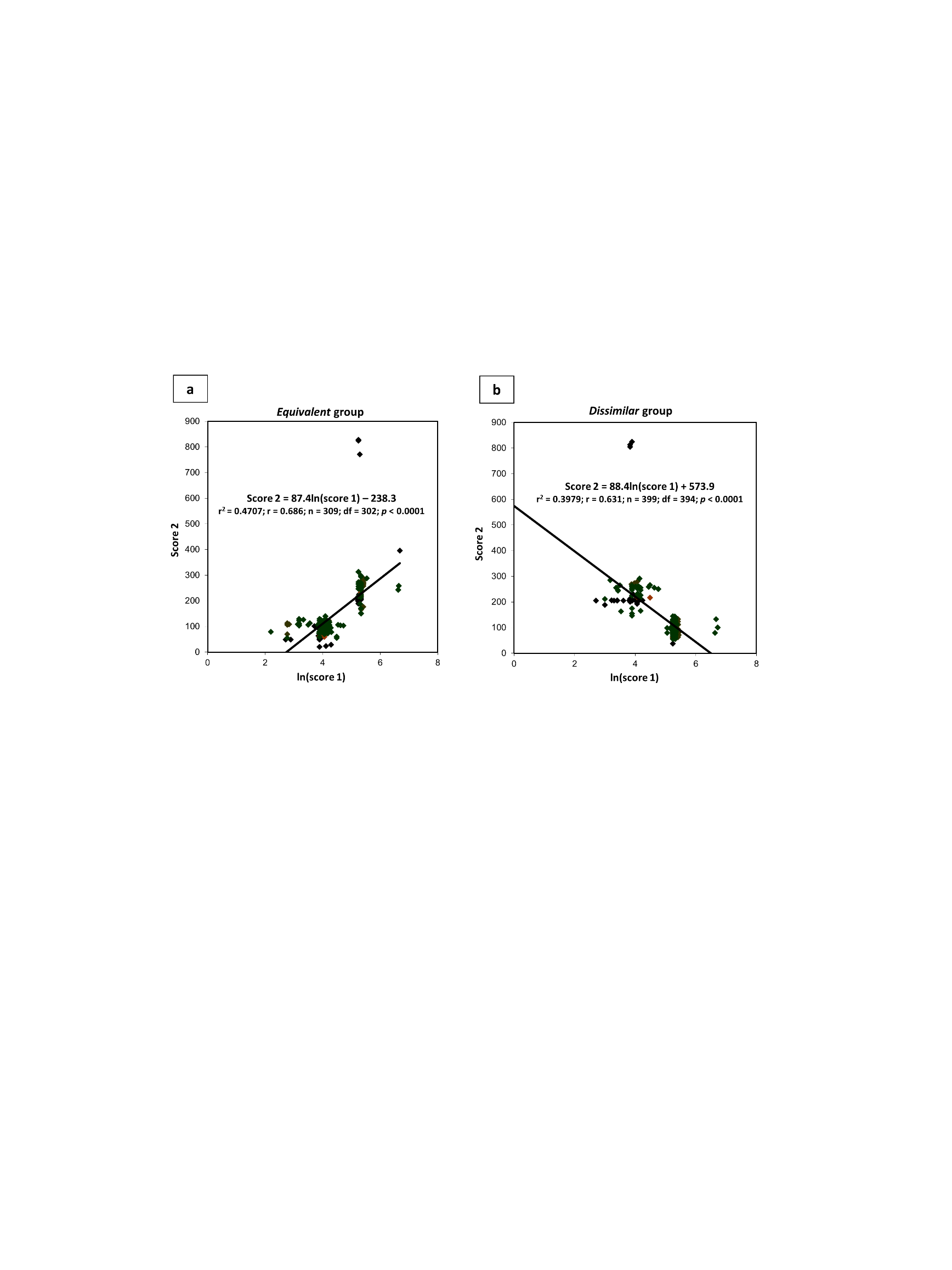


**Supplementary Fig. 2** Logarithmic models comparison between reference set and sets 1, 2 and 3 in *equivalent* (a) and *dissimilar* (b) groups. The reference set is shown in black, set 1 in brown, set 2 in red, and set 3 in green. Supplementary Table 4 shows the individual linear regression models

**
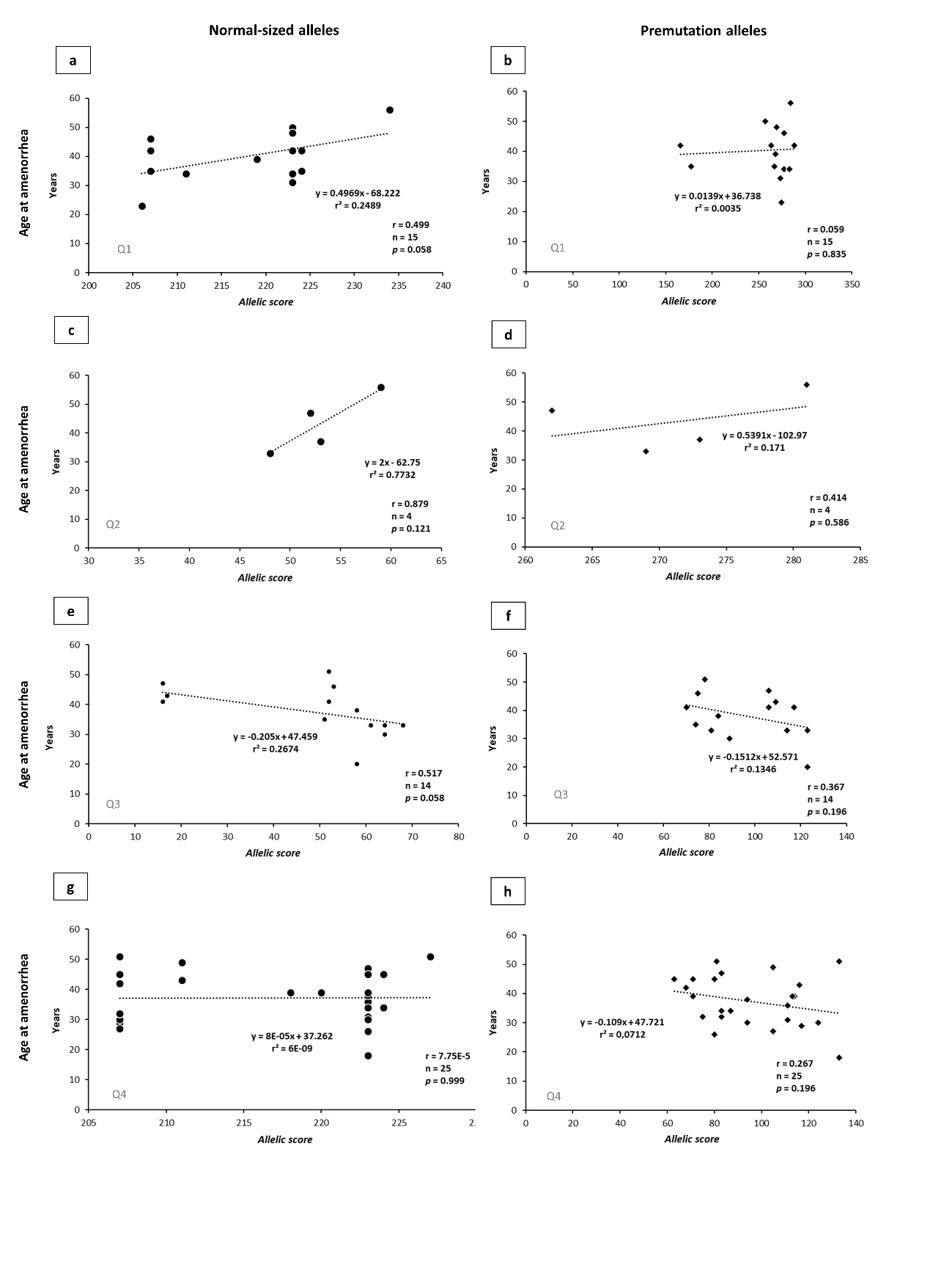
**

**Supplementary Fig. 3** Correlation between the *allelic score* of normal-sized alleles (3a, c, e and g) and premutation (3b, d, f and h) allele with age at amenorrhea in samples from set 2. Qn – represents the four quadrants obtained after *allelic scores* combination (see Fig. 1 - lozenges). No statistically significant correlations were found (all *p* > 0.05)
